# pSuc-EDBAM: Predicting lysine succinylation sites in proteins based on ensemble dense blocks and an attention module

**DOI:** 10.1186/s12859-022-05001-5

**Published:** 2022-10-31

**Authors:** Jianhua Jia, Genqiang Wu, Meifang Li, Wangren Qiu

**Affiliations:** 1 Computer Department, Jingdezhen Ceramic University, Jingdezhen, 333403 China; 2 Computer Department, Nanchang Institute of Technology, Nanchang, 330044 China

**Keywords:** Lysine succinylation, Post-translational modifications, Feature maps, Ensemble dense blocks, Feature learning, An attention module

## Abstract

**Background:**

Lysine succinylation is a newly discovered protein post-translational modifications. Predicting succinylation sites helps investigate the metabolic disease treatments. However, the biological experimental approaches are costly and inefficient, it is necessary to develop efficient computational approaches.

**Results:**

In this paper, we proposed a novel predictor based on ensemble dense blocks and an attention module, called as pSuc-EDBAM, which adopted one hot encoding to derive the feature maps of protein sequences, and generated the low-level feature maps through 1-D CNN. Afterward, the ensemble dense blocks were used to capture feature information at different levels in the process of feature learning. We also introduced an attention module to evaluate the importance degrees of different features. The experimental results show that Acc reaches 74.25%, and MCC reaches 0.2927 on the testing dataset, which suggest that the pSuc-EDBAM outperforms the existing predictors.

**Conclusions:**

The experimental results of ten-fold cross-validation on the training dataset and independent test on the testing dataset showed that pSuc-EDBAM outperforms the existing succinylation site predictors and can predict potential succinylation sites effectively. The pSuc-EDBAM is feasible and obtains the credible predictive results, which may also provide valuable references for other related research. To make the convenience of the experimental scientists, a user-friendly web server has been established (http://bioinfo.wugenqiang.top/pSuc-EDBAM/), by which the desired results can be easily obtained.

## Introduction

It has been discovered that succinylation is a novel protein post-translational modification, which is related to a variety of biological processes, including cancer progression and metastasis, and involves in the life activities such as glucose metabolism and amino acid metabolism through regulating the protease activity and gene expression [1]. Owing to the binding of lysine residues to succinyl group after succinylation, a series of changes have taken place in the protein structure. Furthermore, succinylation changes the charge of lysine residues from + 1 to –1, which further changes the physico-chemical properties of amino acids and enriches protein function [2]. Many related studies have fully displayed that succinylation may regulate multiple metabolic processes of organisms [3, 4], whose abnormalities are closely connected with emergence and development of human diseases, which include inflammation, tumors, cardiometabolic diseases, and so on [5, 6]. It can be seen that the importance of succinylation is self-evident, which has also attracted the atteintion of many researchers at home and broad [7].

Currently, a variety of biological experimental approaches have been come up with identifying succinylation sites, for instance, high-performance liquid chromatography assays, mass spectrometry, liquid chromatography-mass spectrometry, and so on [8]. In my opinion, these approaches are both costly and inefficient. Therefore, it is urgent to solve the shortcomings of biological experimental approaches by exploring a novel approach.

In recent years, thanks to the wide application of machine learning, a host of researchers have applied it to identify succinylation sites to solve the weakness of biological experimental approaches [9, 10]. Hasan et al. reviewed the latest advances regarding the current predictors, datasets, and online resources, which provided a useful guideline for developing effective succinylation site prediction tools [11]. Tasmia et al. updated the predictors, datasets, and online resources metioned in this review according to the development in recent years [12]. Xu et al. [13] built a succinylated site predictor based on SVM called iSuc-PseAAC in 2015, but the true distribution of the dataset is not fully taken into account. Jia et al. constructed some predictors including pSuc-Lys [14] and iSuc-PseOpt [15] in 2016; however, these classifiers ignored some important sequence information. Hasan et al. [16] built a predictor called SuccinSite based on the random forest (RF). In 2018, Dehzangi et al. [17] constructed the SSEvol-Suc predictor, which combined PSSM and the secondary structure with the AdaBoost by graph double-byte mapping, which is remarkably superior to previous predictors. Hasan et al. [18] constructed GPSuc by using logical regression (LR) combined with the output of different RF scores. Yosvany et al. [19] proposed a SVM-based predictor named Success with combining the structure and evolution information of amino acids with double-stranded maps. Zhu et al. [20] developed a RF-based predictor named Inspector combined with some sequence feature encoding schemes in 2020. To reduce the computational complexity, Zeng et al. [21] proposed a computational method named iSuc-ChiDT in 2022. Obviously, these approaches adopt manual feature selection, but it is difficult to find useful potential information [22]. Therefore, it is very necessary to explore a novel predictor which can automatically learn features to predict succinylation sites.

Along with the deepening of study, we found that deep learning (DL) can effectively overcome the shortcomings of the above problems, and can automatically learn useful features from the dataset. In 2020, Ning et al. [23] constructed the predictor named HybridSucc integrating a variety of information features, and adopts the penalized logistic regression algorithm and deep neural network (DNN) to make the model optimized. Thapa et al. [24] created a DL-based predictor called DeepSuccinylSite. Huang et al. [25] introduced long short-term memory (LSTM) and convolution neural network (CNN) into DL methods in 2021. These predictors enrich the applications of DL in succinylation site prediction.

Based on the above review, we proposed a novel predictor using ensemble dense blocks and an attention module, called as pSuc-EDBAM. pSuc-EDBAM used one hot encoding to extract the initial protein sequence feature maps, and generated the low-level feature maps through 1-D CNN. Afterward, ensemble dense blocks [26] were adopted to capture the advanced features from the initial features. In addition, an attention module was used to evaluate the importance degrees of different protein sequence features, make every feature map weighted, and then improve the network abstraction ability to predict potential succinylation sites. The features were then matched with the softmax classifier to go on succinylation site prediction. To further illustrate the performance of pSuc-EDBAM, we performed ten-fold cross-validation on the training dataset and independent test on the testing dataset. According to experimental results, our model has yielded promising results and is superior to the existing predictors. The prediction and potential succinylation sites further show that pSuc-EDBAM is a powerful predictor for predicting unknown succinylation sites.

The main contributions of the paper are summarized below: (1) An effective and novel predictor was proposed based on ensemble dense blocks and an attention module for succinylation sites prediction, called as pSuc-EDBAM. (2) An improved attention module was introduced into the prediction of succinylation sites, which improved the prediction ability of succinylation sites. (3) In this paper, our model is simple and easy to use, and features are automatically learned on the basis of feature map extracted by one hot encoding, which greatly improves the ability of succinylation site prediction. (4) The model built in this paper can broaden the thinking of other researchers and do better research. (5) A web-server has been provided at http://bioinfo.wugenqiang.top/pSuc-EDBAM/, by which the desired results may be easily obtained.

## Materials and methods

In binary classification-based lysine succinylation site prediction studies, we labeled each potential site as a succinylated site or a non-succinylated site [27]. In particular, we extracted a protein sequence with length *L* = 2*r* + 1 with lysine (K) as the center, where *r* represents amino acid residues on each side. Firstly, we converted the input into numerical vectors by an encoding method, and then we trained pSuc-EDBAM based on the benchmark training dataset. Finally, its performance was evaluated by comparing it with other existing predictors.

### Benchmark dataset

The benchmark dataset was gathered from the UniProtKB/Swiss-Prot database [28] and NCBI protein sequence database from Ning et al. [29]. In order to reduce the model deviations owing to the sequence homology, we used CD-HIT [30] to remove redundant sequences, and set the threshold to 0.3. Finally, 2322 proteins were retained as our final benchmark dataset, containing 5009 experimentally verified succinylation sites and 53,542 non-succinylation sites. To conveniently compare with other existing methods, 124 proteins were randomly separated from 2322 proteins as an independent testing dataset, and the remaining proteins were used as a training dataset. Table 1 lists the specifies of the benchmark dataset. In order to facilitate further research by researchers, the benchmark dataset can be easily obtained from https://github.com/wugenqiang/pSuc-EBDAM/tree/main/dataset.

**Table 1:** The specifies of the benchmark dataset

Original dataset	Number of proteins	Positive site	Negative site
Training dataset	2198	4755	50,549
Testing dataset	124	254	2977

We adopted Chou's peptide formulation [31], each protein sequence can be defined as Eq. (1).

1 $${P}_{\delta }(K)={R}_{-\delta }{R}_{-(\delta -1)}\cdots {R}_{-2}{R}_{-1}K{R}_{+1}{R}_{+2}\cdots {R}_{+(\delta -1)}{R}_{+\delta }$$

where *K* denotes the lysine and δ denotes an integer, ${R}_{-\delta }$ denotes the $\delta\mathrm{th}$ amino acid residue to the left of *K*, and ${R}_{+\delta }$ denotes the $\delta\mathrm{th}$ amino acid residue to the right of *K*, so that ${P}_{\delta }\left(K\right)$ can define each protein sequence as two classes as shown in Eq. (2).

2 $${P}_{\delta }(K)\in \left\{\begin{array}{ll}{ P}_{\delta }^{+}(K), &if\, the \,center\, is\, a\, succinylation\, site\\ {P}_{\delta }^{-}(K), &otherwise\end{array}\right.$$

It is not difficult to find that some protein sequences have some non-standard residues, such as "X", we adopted a better approach from Jia's paper [14], which made this part of amino acid residues filled via mirroring image, as defined in Eqs. (3) and (4).

(A) The mirror image of carbon-terminus
  3 $${R}_{+\delta }{R}_{+(\delta -1)}\cdots {R}_{+2}{R}_{+1}\underset{K}{\iff }{R}_{+1}{R}_{+2}\cdots {R}_{+(\delta -1)}{R}_{+\delta }$$
(B) The mirror image of nitrogen-terminus
  4 $${R}_{-\delta }{R}_{-(\delta -1)}\cdots {R}_{-2}{R}_{-1}\underset{K}{\iff }{R}_{-1}{R}_{-2}\cdots {R}_{-(\delta -1)}{R}_{-\delta }$$

The mirror image of carbon-terminus is on the left of "$\Leftrightarrow$" in Eq. (3), and the mirror image of nitrogen-terminus is on the right of "$\Leftrightarrow$" in Eq. (4); while the original sequence is on the other side, with "$\Leftrightarrow$" indicating the mirror image and *K* representing the lysine.

### Sequence feature extraction via one hot encoding

One hot encoding is a common feature extraction method to reflect the types and positions of amino acid residues directly, which has been maturely applied to the process of protein feature extraction [32]. In this study, we applied this method to obtain feature maps from the protein sequences for further research. We listed the 20 amino acid residues in alphabetical sequence as ACDEFGHIKLMNPQRSTVWY and enforced the following rule: the *i*th amino acid residue was labeled as 1 in the *i*th position and 0 in the other positions, such as amino acid residue D was coded as 00100000000000000000, and then the protein sequence of length *L* can be converted into $L\times 20$ dimensional feature vector.

### Model construction

**Fig. 1:**
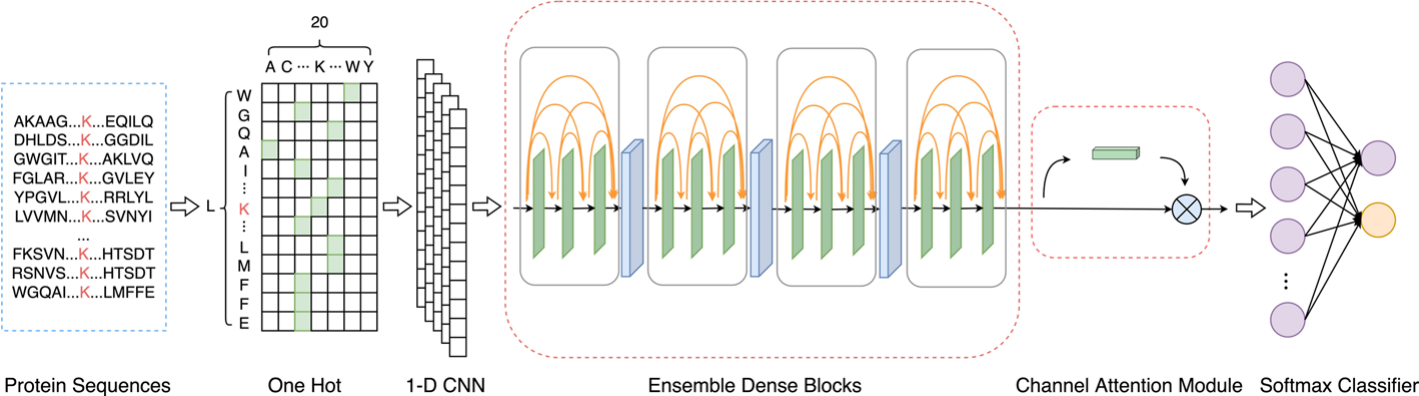
The framework of the pSuc-EDBAM model

We constructed a model to learn the deeply hidden features of succinylation sites efficiently in this study, called as pSuc-EDBAM. In this pSuc-EDBAM model, ensemble dense blocks [26] were adopted to obtain the advanced features. Afterward, we introduced an attention module to evaluate feature importance degrees. Eventually, the advanced features were input into the softmax classifier to predict succinylation sites. The framework of pSuc-EDBAM is shown in Fig. 1.

### 1-D CNN

The convolution neural network (CNN) is a common feed-forward network, which was proposed by LeCun et al. [33, 34] and has some notable advantages such as parameter sharing and local connectivity. In 1-D CNN, the CNN kernel moves in one direction to extract the protein sequence features, and the dimensions of input and output data are both two-dimensional, mainly used for sequence model, while in 2-D CNN, the CNN kernel moves in two directions to extract the protein sequence features, and the dimensions of input and output data are both three-dimensional, mainly used for image data [35].

Here, we used 1-D CNN to extract low-level features. Suppose a discrete sequence is $\alpha =[{\alpha }_{1}, {\alpha }_{2}, \ldots ,{\alpha }_{n}]$, and the convolution kernel is $\beta =[{\beta }_{1},{\beta }_{2},\ldots , {\beta }_{m}]$. The 1-D CNN of $\alpha$ and $\beta$ is expressed as Eq. (5).

5 $$\alpha *\beta =\left[\sum_{i=1}^{m}{\alpha }_{jd+i-1}{b}_{i}\right], \quad j = 1, 2, \ldots , k$$

where $d$ represents the stride of convolution and $k$ indicates the length of the output sequence features, which is the most integer less than or equal to $\frac{(n-m)}{d}+1$.

After adopting one hot encoding to extract the feature map, we generated the low-level feature map through 1-D CNN, as shown in Eq. (6).

6 $${X}^{0}=\sigma (I*W+b)$$

where $I$ denotes the feature map extracted from one hot encoding, $W$ denotes the weight matrix, $b$ is used to denote the bias term, $\sigma$ is used to denote the exponential linear unit (ELU) activation function [36], and ${X}^{0}$ denotes the low-level feature map generated by the 1-D CNN.

### Ensemble dense blocks for further feature extraction

**Fig. 2:**
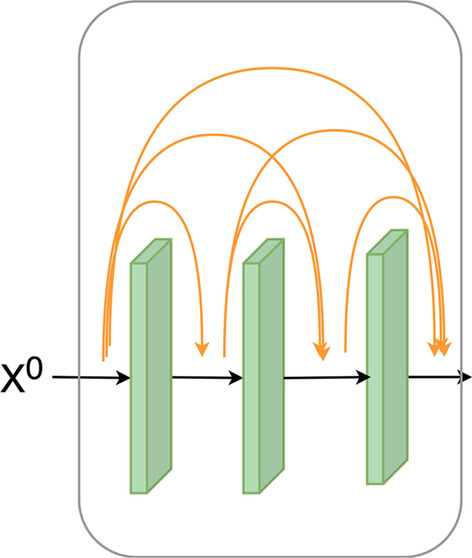
The structure of a dense block

For the sake of extracting the advanced features of succinylation sites, we introduced the ensemble dense blocks, which have been shown to perform higher than traditional CNN. The structure of a dense block is expressed as Fig. 2.

The advanced feature representation of the low-level feature map was extracted by the dense block, which is expressed as Eq. (7).

7 $${X}^{l}=\sigma ([{X}^{0};{X}^{1};\ldots ;{X}^{l-1}]*{W}^{{\prime}}+{b}^{{\prime}})$$

where ${X}^{l-1}$ represents the feature map generated by the ${(l-1)}{\text{th}}$ convolutional layer within the dense block. ${W}^{{\prime}}$ represents the weight matrix, ${b}^{{\prime}}$ denotes the bias term. The output value of the dense block is the concatenation of ${X}^{0}$, ${X}^{1}$, $\ldots$, and ${X}^{l}$.

The next step was to build the transition layer, which is expressed as Eq. (8).

8 $$X=\sigma ([{X}^{0};{X}^{1};\ldots ;{X}^{l}]*{W}^{{\prime\prime}}+{b}^{{\prime\prime}})$$

where ${W}^{\prime\prime}$ denotes the weight matrix, ${b}^{\prime\prime}$ represents the bias term, and $X$ refers to the output value of the transition layer. Afterward, the average pooling was performed on $X$ to reduce the risk of overfitting.

In the study, we integrated four dense blocks. What's more, Eq. (8) is not performed after the fourth implementation of Eq. (7) but replaces it with global average pooling. Ultimately, the advanced feature ${X}^{(seq)}$ was extracted after the above process.

### An attention module for learning feature importance degrees

**Fig. 3:**
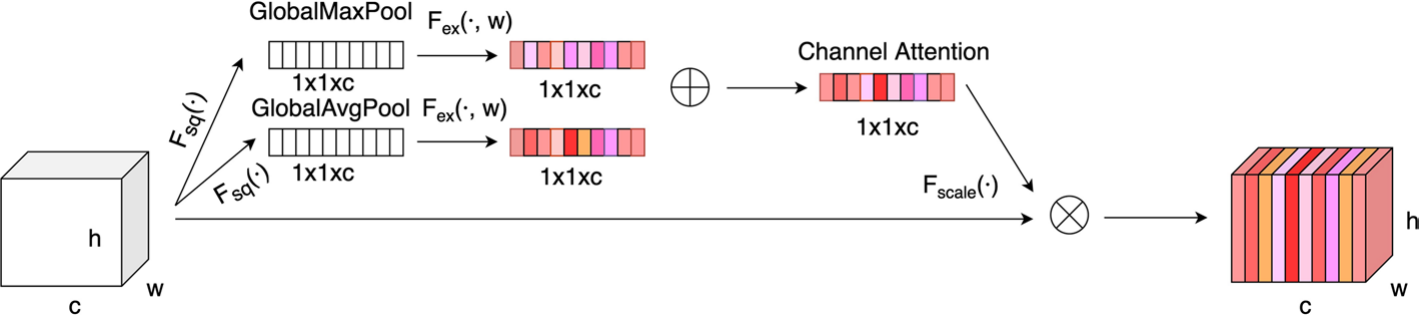
The structure of the channel attention module

From my point of view, different features have different degrees of importance. Consequently, we introduced an attention module to learn feature importance degrees and make every feature map weighted. Here, we proposed the channel attention module, which is implemented via global average pooling, global max pooling, and two fully connected layers, which adds the global max pooling to increase the receptive field of the channel and the importance of learning characteristics more comprehensively based on SE [37] module. The structure of the channel attention module is described in Fig. 3.

For the advanced feature ${X}^{(seq)}$, the channel attention module used global average pooling and global max pooling to squeeze the space information of ${X}^{(seq)}$ into the channel $z$, separately, which respectively used Eq. (9) to get the compressed result ${z}_{avg}$ and ${z}_{max}$.

9 $$z={F}_{sq}({X}^{(seq)})=\frac{1}{w\times h}\sum_{i=1}^{h}\sum_{j=1}^{w}{X}^{(seq)}(i,j)$$

where $w$ is the width of ${X}^{(seq)}$ and $h$ is the height of ${X}^{(seq)}$.

Thereafter, two fully connected layers were adopted to process ${z}_{avg}$ and ${z}_{max}$ respectively to obtain the channel information of ${X}^{(seq)}$ and learn the weight of ${X}^{(seq)}$, as described in Eq. (10), and then got ${s}_{avg}$ and ${s}_{max}$, respectively.

10 $$s={F}_{ex}(z, W) = \sigma ({W}_{2}*\tau ({W}_{1}*z))$$

where $s$ denotes the weight of ${X}^{(seq)}$, $\tau$ means a rectified linear unit (RELU) function and $\sigma$ represents a sigmoid function. ${W}_{1}$ and ${W}_{2}$ are the parameter. To make the weight information of captured every feature map more comprehensive, we added ${s}_{avg}$ and ${s}_{max}$ to get the specific weight information of every feature map, as described in Eq. (11).

11 $$s={s}_{avg}+{s}_{max}$$

Ultimately, the output value of the attention module can be got by scaling ${X}^{(seq)}$ with the activation described in Eq. (12).

12 $${X}^{(seq)}={F}_{scale}({X}^{(seq)}, s)=s\cdot {X}^{(seq)}$$

where ${F}_{scale}({X}^{(seq)}, s)$ indicates that each specific value of ${X}^{(seq)}$ is multiplied by the weight $s$.

### Softmax classifier

On the basis of the advanced features, the softmax classifier was adopted to predict succinylation sites in this study, which received the advanced features as input, and then weighted summation and activation operations are performed to obtain the predicted results of succinylation sites, just as Eq. (13).

13 $$P(y=i|x)=\frac{{e}^{{W}_{i}^{s}*X+{b}_{i}^{s}}}{\sum_{j=1}^{2}{e}^{{W}_{j}^{s}*X+{b}_{j}^{s}}}$$

where ${W}_{i}^{s}$ and ${W}_{j}^{s}$ indicate the weight matrices, ${b}_{i}^{s}$ and ${b}_{j}^{s}$ indicate the bias terms, and $x$ denotes the samples. $P(y=i|x)$ refers to the probability that $x$ is predicted to be $i$. Owing to succinylation site prediction may be considered as a problem of the binary classification, therefore $i=0 \text{ or } i =1$.

The decision threshold refers to the decision that converts the prediction probability into the target class. In this paper, the threshold we use is the default value, set to 0.5. When the prediction probability is higher than 0.5 ($P>0.5$), the site is predicted to be succinylated, and when the prediction probability is lower than 0.5 ($P<0.5$), the site is predicted to be non-succinylated.

### Model training

The pSuc-EDBAM was carried out based on Keras 2.8 (https://keras.io/) in this paper, which is a flexible, simple, and Python-based approach. We adopted the cross entropy as the loss function, which is shown in Eq. (14).

14 $$C=-\frac{1}{n}\sum_{j=1}^{n}{y}^{j}lnP({y}^{j}=1|{x}^{j})+(1-{y}^{j})lnP({y}^{j}=0|{x}^{j})$$

where $n$ represents the number of training samples, ${x}^{j}$ indicates *j*th input, and ${y}^{j}$ represents the true label of ${x}^{j}$. The loss function was optimized by Adam optimizer [38], and the parameters were adjusted by the gradient descent method to minimize the loss function.

Additionally, ${L}_{2}$ regularization was adopted to weaken the negative influence of overfitting, we also used dropout [39] and early stopping [40] to further avoid overfitting. The ratio of positive samples to negative samples in our succinylated dataset is 1:11, which is very unbalanced. To weaken the influence of unbalanced dataset, we introduced the class weight and set the class weight ratio of positive samples to negative samples to 11:1. By this means, the pSuc-EDBAM model could improve the influence of positive samples, so as to further improve the recognition rate of succinylation sites.

### Performance evaluation

Four common metrics were considered to evaluate the performance of pSuc-EDBAM reasonably as previously described [41], including sensitivity (Sn), specificity (Sp), accuracy (Acc), and Mathews Correlation Coefficient (MCC) [42], which are defined as Eq. (15).

15 $$\left\{\begin{array}{l} Sp=\frac{TN}{TN+FP}\\ Sn=\frac{TP}{TP+FN}\\ Acc=\frac{TP+TN}{TP+TN+FP+FN}\\ MCC=\frac{TP\times TN-FP\times FN}{\sqrt{\left(TP+FP\right)\times \left(TP+FN\right)\times \left(TN+FP\right)\times \left(TN+FN\right)}}\end{array}\right.$$

where TP, TN, FP, and FN denote true positive samples, true negative samples, false positive samples, and false negative samples, respectively. Sn was adopted to measure the proportion of predicting succinylation sites correctly, Sp measured the proportion of predicting non-succylation sites correctly, and Acc revealed the proportion of predicting sites correctly. When the distributions of samples are very imbalanced, MCC was considered to be the more noteworthy measure because it can more accurately reflect the quality of the model [43]. In general, the value of MCC is –1, indicating that the prediction of succinylated sites is completely wrong; the value of MCC is 0, indicating that the prediction effect of succinylation sites is not better than that of random prediction, and the value of MCC is + 1, meaning that the prediction of succinylation sites is completely correct.

What's more, the receiver operating characteristic (ROC) curve was adopted to reveal the performance of the model. On this basis, we introduced the area under the ROC curve (AUC) to further intuitively explain the performance of the model. The higher the AUC, the better the overall performance of the model. ten-fold cross-validation was adopted to evaluate the robustness of pSuc-EDBAM and independent test was used to compare the performance of pSuc-EDBAM with the existing predictors.

## Results and discussion

### Select the best window size of succinylation sites

**Fig. 4:**
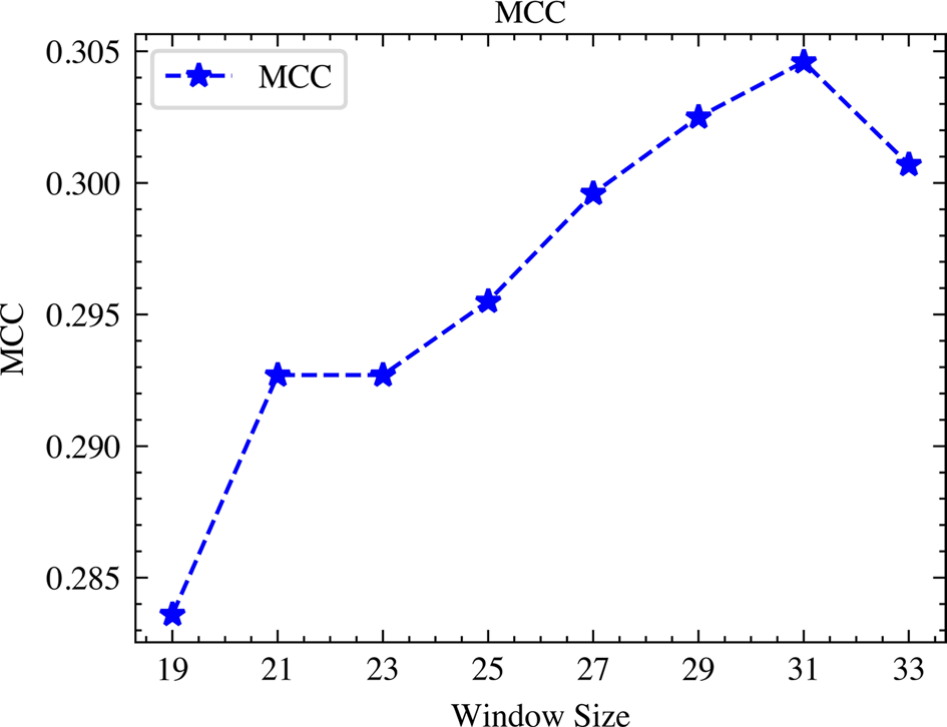
The values of MCC under different window sizes are based on the training dataset

The size of the protein sequence directly determines the feature representation learned by the model, so the selection of its value has an important influence on the succinylation site prediction. To gain the best window size of the succinylation sites of the pSuc-EDBAM model, it is especially necessary to make full use of the automatic and efficient feature extraction of CNN. Based on the training dataset, we chose 19, 21, 23, 25, 27, 29, 31, and 33 as the window size, tested each window size by ten-fold cross-validation, and then averaged the experimental results. The experimental results are shown in Fig. 4. MCC value reaches maximum when the window size is 31, which indicates that 31 is the best window size of the succinylation sites of the pSuc-EDBAM model.

### Sequence analysis of succinylation sites

**Fig. 5:**
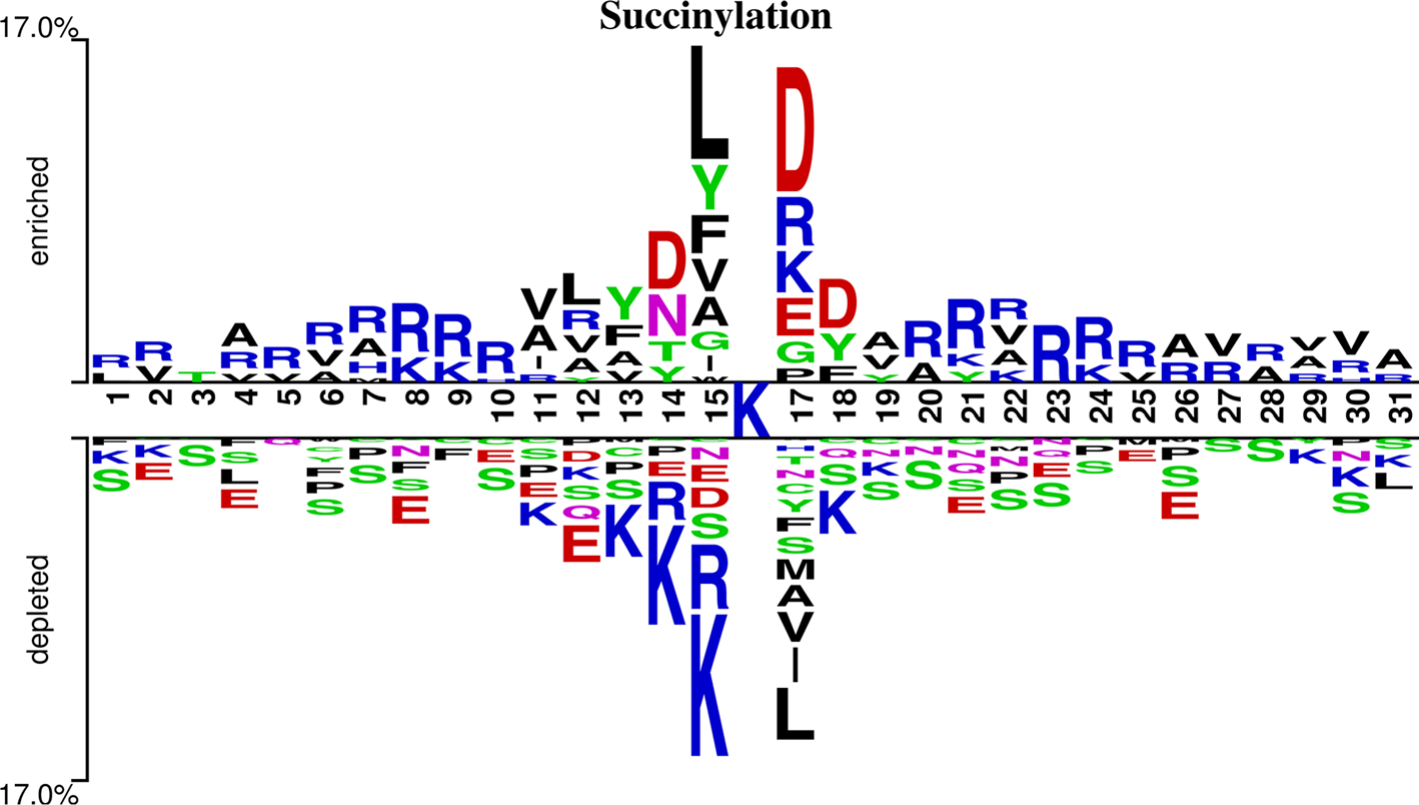
A two-sample logo of succinylation sites against non-succinylation sites with *L* = 31

This investigation analyzed the frequency of occurrence of 30 amino acid residues surrounding the succinylation site on fragment protein sequences to find the potential consensus motifs. Two Sample Logo [44] is an effective tool to find statistically noteworthy differences in position-specific symbol compositions between the succinylated and non-succinylated sites. To better distinguish succinylation sites and non-succinylation sites in the samples, we used Two Sample Logo to analyze the protein sequences and looked into the frequency and position differences of 20 amino acid residues near the succinylation sites and non-succinylation sites, as described in Fig. 5.

In this study, the lysine (K) amino acid residue was placed in the middle of the fragment protein sequences, and positions of the flanking amino acid residues were shown in the range from 1 to 31. The comparison between 4755 succinylation sites and 50,549 non-succinylation sites in Fig. 5 demonstrates that the amino acids including alanine (A), asparagine (D), leucine (L), arginine (R), and valine (V), had the highest ratios around the succinylation site, glutamicacid (E), lysine (K), serine (S) appeared more frequently around the non-succinylation site. Therefore, we concluded that the frequencies and positions of amino acid residues around the succinylation site and the non-succinylation site are different, and the analysis showed that the distance among amino acid characteristics in a sequence plays a vital role in distinguishing between succinylation sites and non-succinylation sites.

### Performance of pSuc-EDBAM on the training dataset

To analyze the performance of pSuc-EDBAM, we conducted ten-fold cross-validation on the training dataset shown in Table 1. As described in Table 2, we find that the values of Sn, Sp, Acc, and MCC on the training dataset are very stable, and the fluctuation is relatively small, indicating that our proposed model has a certain stability, and effectively avoids the problem of overfitting. Therefore, the pSuc-EDBAM model has good performance on the training dataset.

**Table 2:** Performance of pSuc-EDBAM on the training dataset

Fold times	Sn (%)	Sp (%)	Acc (%)	MCC
1	77.10	73.16	73.49	0.3043
2	76.68	74.44	74.63	0.3130
3	81.30	72.01	72.81	0.3190
4	76.26	72.36	72.70	0.2928
5	77.52	73.17	73.54	0.3069
6	73.89	72.74	72.84	0.2819
7	75.16	76.48	76.37	0.3224
8	79.79	69.73	70.60	0.2919
9	79.16	71.41	72.08	0.3015
10	72.21	77.25	76.82	0.3122
Mean ± STD	76.91 ± 2.60	73.28 ± 2.15	73.59 ± 1.80	0.3046 ± 0.0122

**Fig. 6:**
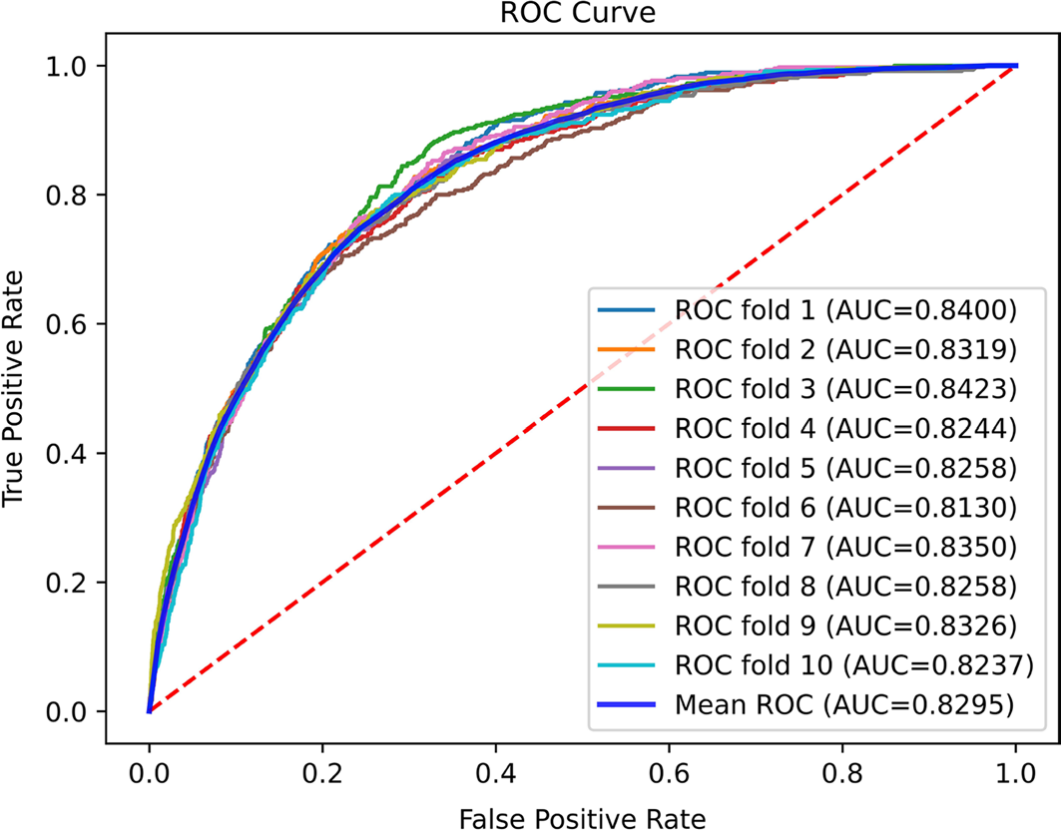
The receiver operating characteristics (ROC) curve for pSuc-EDBAM on the training dataset. AUC denotes the area under the ROC curve

Figure 6 shows the ROC curve of the pSuc-EDBAM on the training dataset, and the mean of AUC is 0.8295. The result indicates that our proposed pSuc-EDBAM has notable advantages including better stability.

### Comparison with the existing predictors

To certify the effectiveness of our predictor named pSuc-EDBAM, we conducted independent test on the same independent testing dataset to objectively compare pSuc-EDBAM with existing predictors. Eight existing predictors were considered, including SuccinSite [16], SuccinSite2.0 [45], Success [19], pSuccE [29], GPSuc [18], Inspector [20], iSuc-ChiDT [21], and iSuccLys-BLS [46]. The details are shown in Table 3. SuccinSite was established based on RF which was trained with three combined encodings. SuccinSite2.0 was also constructed through RF, but it took the composition of profile-based amino acid and orthogonal binary features as the training data. Success was constructed by using the structural and evolutionary information of amino acids to extract protein features. PSuccE was another classifier combining multiple features with a feature selection scheme. GPSuc was established based on RF. iSuc-ChiDT was proposed to identify succinylation sites using statistical difference table encoding and the chi-square decision table classifier. iSuccLys-BLS was constructed using a broad learning system (BLS), which optimized the imbalanced training dataset using randomly labeling samples. The novel deep learning-based predictor pSuc-EDBAM was proposed in this paper based on ensemble dense blocks and an attention module, which adopted one hot encoding to capture the protein sequence feature.

**Table 3:** Performance comparison of pSuc-EDBAM with other existing predictors on the independent testing dataset

Predictor	Sn (%)	Sp (%)	Acc (%)	MCC
SuccinSite	37.10	88.20	84.20	0.1990
SuccinSite2.0	45.40	88.20	84.80	0.2610
Success	14.20	86.80	81.10	0.0700
PSuccE	37.50	88.60	84.50	0.2040
GPSuc	49.90	88.30	85.30	0.2960
Inspector	69.30	71.70	71.50	0.2380
iSuc-ChiDT	70.47	66.27	68.30	0.2050
iSuccLys-BLS	72.30	68.90	69.20	0.2340
pSuc-EDBAM	75.59	74.13	74.25	0.2927

The ratio of positive samples to negative samples in the independent testing dataset studied in this paper is about 1:11, and the dataset is extremely unbalanced. In the study of this kind of unbalanced data, Sn and MCC are the main indicators to consider, and the improvement of these two indicators is particularly important. As can be seen in Table 3, based on the independent testing dataset, the pSuc-EDBAM gained higher values of Sn, which is 3.29% higher than the current best predictor named iSuccLys-BLS [45]. We know that Sn means the proportion of all positive samples that are correctly predicted as positive samples, and then evaluates the predictor's performance in predicting positive samples. Therefore, the novel predictor pSuc-EDBAM with the higher Sn value is more significant and practical for predicting succinylation sites. We find that the earliest predictors have high Sp and low Sn, such as SuccinSite, SuccinSite2.0, Success, pSuccE, and GPSuc. This is because these predictors did not take into account the imbalance of the data or find a reliable method to solve the problem of unbalanced data, which caused the recognition of these predictors to the positive samples was not obvious. When noticing the unbalanced distribution of the dataset, the values of Sn and Sp would tend to be balanced. It means that the predictor taking into account the data distribution is of more practical significance, such as Inspector, iSuc-ChiDT, iSuccLys-BLS, and our proposed predictor called pSuc-EDBAM. It is found that our predictor is significantly superior to Inspector, iSuc-ChiDT, and iSuccLys-BLS in all metrics.

**Fig. 7:**
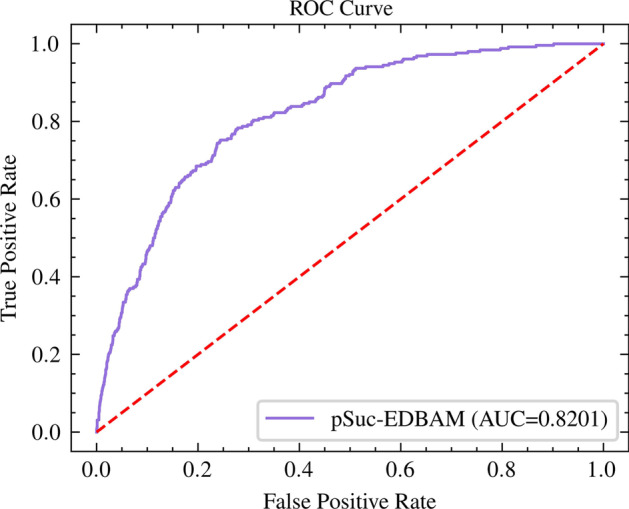
The receiver operating characteristics (ROC) curve for pSuc-EDBAM on the independent testing dataset. AUC denotes the area under the ROC curve

When AUC is nearer 1, the performance of predictor is better. Figure 7 shows the ROC curve of the pSuc-EDBAM on the independent testing dataset, and AUC is 0.8201. The result indicates that our proposed novel predictor has more advantages and better stability. Therefore, it is expected that pSuc-EDBAM may be a more representative and meaningful tool in succinylation site prediction.

**Fig. 8:**
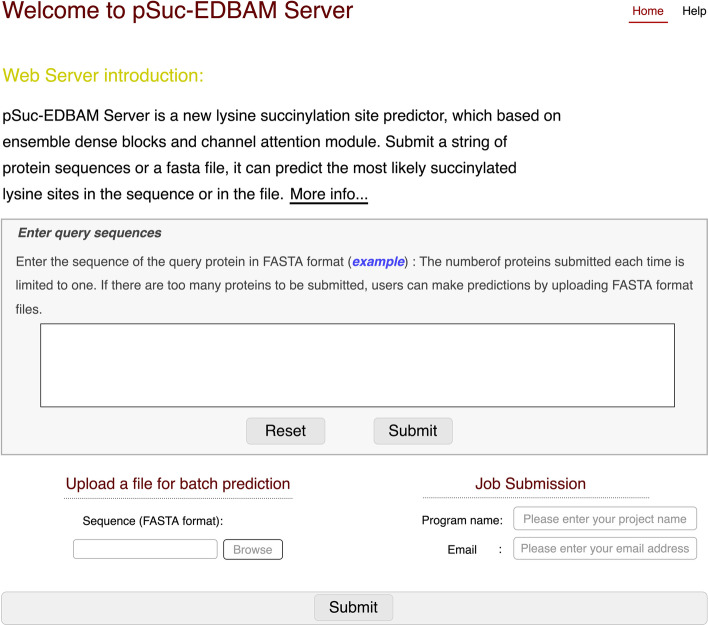
The homepage of the pSuc-EDBAM predictor at http://bioinfo.wugenqiang.top/pSuc-EDBAM/

### Implementation of the pSuc-EDBAM predictor and user guide

An effective predictor can be beneficial for researchers to study the protein succinylation sites. In this study, an open online web-based predictor named pSuc-EDBAM is designed to analyze protein succinylation sites efficiently, which can be accessed at http://bioinfo.wugenqiang.top/pSuc-EDBAM/. To make the convenience of most researchers, we supply a user guide below:

- *Step 1*: Open the homepage of the pSuc-EDBAM predictor as described in Fig. 8. You can click on the web page button such as "Help" or "More info…" to look up the profile of the pSuc-EDBAM web server.
- *Step 2*: Enter a single protein sequence according to the prompt, which is required to be in FASTA format. Click on "example" button and you can see the example of a protein sequence in FASTA format.
- *Step 3*: Click on "Submit" button after inputing the protein sequence and you can obtain the predicted results of the succinylation sites.
- *Step 4*: The users of the pSuc-EDBAM predictor can upload files by the "Browse" button and these files must be in FASTA format. Then you need to leave the project name and your email address so that we can send the predicted results of the succinylation sites to you in a timely manner.

## Conclusion

In this paper, we proposed a novel predictor called pSuc-EDBAM for succinylation site prediction, which used ensemble dense blocks and an atteintion module. The efficiency of the pSuc-EDBAM was demonstrated by ten-fold cross-validation on the training dataset and independent test on the testing dataset.

Although pSuc-EDBAM has shown strong robustness in predicting succinylation sites, it still has some weakness. In the course of continuous learning, we have learned that the deep learning is regarded as a black box, which may not be explained in biological processes [47]. In the following work, we will take biological interpretation into consideration and apply more effective attention modules including the convolutional block attention module (CBAM) [48], external attention (EA) [49], and so on, through which will have more meaningful gains in the following experiments.

Considering these together, although further improvement should be conducted as new dataset are available, the pSuc-EDBAM will provide useful information for further experimental manipulation. With the upgrading of technology and the rapid development of proteomics research technology, new research approaches emerge in endlessly, which will bring great convenience to the medical field. It is helpful to further reveal the regulation mechanism of succinylation and provide new ideas for the biomedical research.

## Data Availability

The dataset and source code used in this study can be easily derived from https://github.com/wugenqiang/pSuc-EDBAM.
